# Thyme honey reduced hyperglycemia in male rats subjected to chronic unpredictable mild stress: Possible involvement of GLUT4 protein and circulating irisin

**DOI:** 10.22038/ajp.2025.25486

**Published:** 2025

**Authors:** Maedeh Ghasemi, Forouzan Sadeghimahalli, Hassan Jamali, Azadeh Yazdi, Mohammad Reza Seyedi Moqadam

**Affiliations:** 1 *Department of Physiology, School of Medicine, Isfahan University of Medical Sciences, Isfahan, Iran*; 2 *mmunogenetics Research Center, School of Medicine, Mazandaran University of Medical Sciences, Sari, Iran*; 3 *Department of Physiology, School of Medicine, Mazandaran University of Medical Sciences, Sari, Iran*; 4 *Department of Medicine, Najafabad Branch, Islamic Azad University, Najafabad, Iran*; 5 *Clinical Research Development Center, Najafabad Branch, Islamic Azad University, Najafabad, Iran*; 6 *Department of Toxicology, School of Pharmacy, Mazandaran University of Medical, Sari, Iran*

**Keywords:** Thyme honey, GLUT4, Irisin, Hyperglycemia, Chronic stress

## Abstract

**Objective:**

Chronic stress is a common and fundamental problem in human life all over the world which threatens the health. Stress-induced metabolic disorders are attenuated by natural honey feeding. So, we examined protective impact of thyme honey in the regulation of blood glucose via measuring the expression level of muscle GLUT4 protein in chronic unpredictable mild stressed (CUMS) male rats.

**Materials and Methods:**

Six groups of adult male Wistar rats were designed in this study; control group that received water; unstressed groups that were treated with honey (0.2 and 2 g/kg/day) for 38 days; stressed group that received CUMS for 4 weeks; treated stressed groups that were gavaged by honey (0.2 and 2 g/kg/day) for 38 days (from 10 days before induction of stress until the end of stress period). A day after the experiment period (39^th^ day), in non-fasting status, rats were sacrificed to measure glucose, insulin, irisin, lipid profile at serum level and GLUT4 protein content in muscle tissue via western blotting method.

**Results:**

Honey reduced hyperglycemia induced by CUMS, significantly increased serum irisin and non-significantly increased high-density lipoprotein cholesterol (HDL-c) which were decreased by CUMS, but did not affect other serum lipids and insulin. CUMS down-regulated GLUT4 protein level. Honey feeding (2 g/kg) in stressed rats interestingly increased level of GLUT4 protein and maintained it at a normal level.

**Conclusion:**

Together, it may be concluded that honey administration protects glycemic control system from chronic stress-induced dysregulation via an increase in production of irisin and maintaining the GLUT4 protein levels.

## Introduction

Chronic unpredictable mild stress (CUMS) is a valid animal model of stress that has similarity to depression‐like disorders in human (Su et al., 2016). Stress has many complications such as metabolic disorders. Impaired glucose homeostasis is one of these metabolic complications. Stress-induced hyperglycemia is resulted from altering the physiological mechanisms to control glycemic status (Pereira et al., 2016; Fu et al., 2009; Tamashiro et al., 2011; McCowen et al., 2001; Salimi et al., 2016). One of mechanisms to control blood glucose is insulin secretion from beta-cell of endocrine pancreas in response to glucose. Insulin evokes the entrance of glucose into cells via different glucose transporters which are inserted into plasma membrane (Huang et al., 2001). 

Insulin stimulates glucose absorption by translocation of the insulin-sensitive glucose transporter isoform 4 (GLUT4) from cytoplasm to the plasma membrane in muscle and adipose tissues (Mohammad et al., 2006). Insulin not only induces the GLUT4 exocytosis but also inhibits its endocytosis in target cells (Huang et al., 2001). Insulin resistance is defined as an impairment in insulin signaling pathway and a reduction of GLUT4 and finally a decrease in glucose uptake (Hoehn et al., 2009). The result of a systematic review study showed that there is a positive association between chronic stress (depression) and insulin resistance (Kan et al., 2013). In another study, induction of chronic stress for 28 days could evoke insulin insensitivity and hyperglycemia through down-regulation of GLUT4 levels in skeletal muscles (Morakinyo et al., 2016). 

Several factors are involved in insulin sensitivity improvement such as irisin which is secreted by muscle and adipose tissues. Irisin as an adypomyokin increases glucose uptake by improving insulin signaling. It increases the translocation of GLUT4-contaning vesicles from intracellular component to cell surface in skeletal muscle (Yang et al., 2015; Xin et al., 2016). It has been shown that irsin increased lipid metabolism as well as glucose utilization by modulating the adenosine monophosphate (AMP)-activated protein kinase (AMPK) signaling pathway in myocytes of diabetic mice (Xin et al., 2016). Chronic stress produces lipid metabolism disorders. In a study conducted by Tang et al. (2015), CUMS for 12 weeks could elevate the blood level of total cholesterol (TC), triglyceride (TG), low-density lipoprotein cholesterol (LDL), and decreased high-density lipoprotein cholesterol (HDL) (Tang et al., 2015b). The same results were observed in other studies (Wang et al., 2014; Zeeni et al., 2013). 

For centuries, natural products such as honey have been consumed by human for remedy of many diseases and their complication. Natural honey has various medicinal benefits such as antioxidant, hypoglycemic, anti-lipidemic, anti-inflammatory (Al-Waili, 2004; Erejuwa et al., 2012; Ramli et al., 2018) and other effects. Honey as a sweet product of honeybee, is composed of many substances, mainly carbohydrate including fructose and glucose, protein, water, enzymes, amino acids, vitamins, minerals and polyphenols (Ahmad et al., 2020). There are many animal and human studies showing effect of honey on glycemic regulation and insulin resistance in diabetic status (Erejuwa et al., 2012; Erejuwa et al., 2010; Mazruei Arani et al., 2019), but a few researches studied the effect of honey on metabolic disorders induced by chronic stress. For example, it has been documented that honey feeding in rats subjected to chronic noise stress for 8 weeks, reduced hyperglycemia and hyperlipidemia in serum and oxidative markers in brain tissue as compared to control group (Arabmoazzen et al., 2015). Nowadays, presence of various stressors in human life is common. Metabolic outcomes following chronic stress are the most important risk factors to develop type 2 diabetes and its prevention is very important for researchers. Therefore, the use of honey as the most ancient natural substance in stressful life of today’s world, can be useful in preventing hyperglycemia which is produced by chronic stress. So, the present study investigated the protective effect of thyme honey on glucose homeostasis in male rats subjected to chronic unpredictable stress.

## Materials and Methods

### Animal

Forty eight male Wistar rats (BW; 200-250 g) were housed in polyethylene cages in standard condition (temperature 22+3°C) with a 12 hr-light/dark cycle. Rats had free access to water and food. Rats were randomly divided into 6 groups (n=8); control group, two honey groups that received honey only (0.2 and 2 g/kg) for 38 days, CUMS group that received just vehicle (water as honey solvent) and 2 CUMS + honey groups that received honey (0.2 and 2 g/kg/day) for 38 days (10 days before stress until the end of the stress period. Stress duration was 28 days). CUMS was induced for 4 weeks with or without honey treatments. Honey was consumed by rats via intragastric gavage (0.2 and 2 g/kg, once a day) for 38 consecutive days. The doses of honey were selected based on previous studies (Rafiee Sardooi et al., 2021; Mehranfard et al., 2020). All experimental procedures were approved by the local ethics committee of the Mazandaran University of Medical Sciences, Sari, Iran (IR.MAZUMS.REC.1398.6987, IR.MAZUMS.REC.1398.6868). 

### Experimental procedures

**Figure 1:**
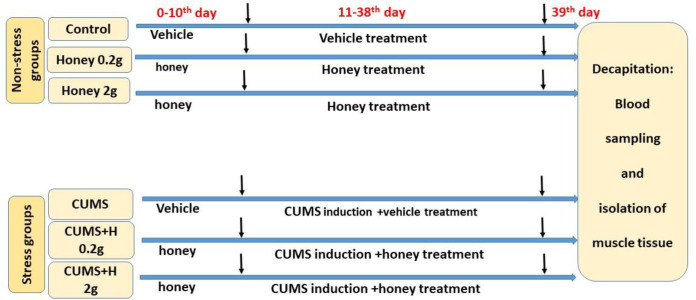
The experimental procedure timeline

A schematic representation of timeline of experimental procedures is shown in Figure 1. The treatment groups received orally thyme honey (0.2 and 2 g/kg/day, Organization of agriculture- Jahad-Alborz, Dehdaz, Iran) or vehicle (water) for 38 consecutive days from 10 days before CUMS induction to the end of the experiment. Gavage was done between 8:00-9:00 am every morning 30 min before stress induction. The day after the last session of stress induction, all rats in non-fasting status were decapitated after deep anesthesia (Ketamine 100 mg/kg + **xylazine** 10 mg/kg) to collect blood samples and extract muscles tissues. Analysis of the components of honey was done using technique/analyzing apparatus of Hourtash Lab, Isfahan (Figure1).

### Induction of chronic unpredictable mild stress

The procedure of chronic unpredictable mild stress was done according to a previous study (Mehranfard et al., 2020). Briefly, for induction of CUMS, rats were subjected to various mild stressors for 28 days. These stressors consisted of tail pinch (1 min, 1 cm from the distal portion of the tail), moist bedding (300 ml of water was added to 300 g sawdust bedding), 40°cage tilt (24 hr), water deprivation (10–12 hr), food deprivation (18 hr), physical restraint (45 min) and overnight lighting. Selection of stressors was randomly and their sequence was altered every week. Every day only one stressor was induced (between 09:00 and 11:00 am). Animals of all groups were weighed every 5 days during CUMS period. 

Unstressed animals were kept in their home cages in a separated room during stress period. 

Chronic unpredictable mild stress in rodents is one of the most commonly employed preclinical models used to understand the onset and the progression of depressive-like state and has been well characterized at the behavioral, physiological, cellular, and molecular levels. When exposed to CUMS, rats display signs of depressive and anxiety-related behaviors (delayed body weight gain, reduction in sucrose solution consumption, increased anxiety in the elevated plus maze and disturbances in the pattern of corticosterone secretion). Because this model is confirmed by the fact that CUMS leads to a decrease in sucrose intake and sucrose preference in animals, a pilot study of sucrose intake test (as described by Rafiee Sardooi et al., 2021) was conducted and results confirmed the induction of CUMS model (reduced sucrose intake). The results of test (data not shown) are presented as supplemental data.

### Blood sampling

At the end of the experimental period, animals of all groups were sacrificed under deep anesthesia (Ketamine 100 mg/kg + **xylazine** 10 mg/kg), and then the rat was taken from its cage and euthanasia applied as soon as possible. After decapitation, blood samples were taken and centrifuged. Eppendorfs containing serum were stored in -80°C to measure glucose, insulin, irisin and lipid profile (Kadirvelu and Gurtu, 2013; Mohd Ramli et al., 2021). 

### Serum biochemical assay

The serum glucose level was detected using the glucose oxidase method (Pars Azmoon, Iran), insulin using a rat insulin ELISA kit (ZelBio, Germany), irisin using a rat irisin ELISA kit (ZelBio, Germany), LDL, HDL, TG and cholesterol using IFCC (International Federation of Clinical Chemistry and Laboratory Medicine) method (ELISA kits; Pars Azmoon, Iran)

### Western blot

After 28 days, CUMS rats were euthanized under deep anesthesia of CO2 inhalation, and then skeletal muscle tissues (gastrocnemii) were removed. All the samples were homogenized using lysis buffer containing Tris-HCl (pH 8), Ethylenediamine tetraacetic acid (EDTA), NaCl, sodium deoxycholate, Sodium dodecyl sulfate (SDS), protease inhibitor cocktail and NP40 (1%)) Triton. The protein level of the lysate was assessed by Bradford protein assay. An equal volume of each protein sample was electrophoresed on 10% SDS-PAGE gels and transferred to polyvinylidene fluoride (PVDF) membranes. Following blocking the PVDF membranes with skim milk (2% non-fat dry milk in Tris-buffered saline, 0.1% Tween 20 or TBS-T buffer), the membranes were incubated at 4°C overnight with the primary antibodies against rat anti-GLUT4 (H-61): sc-7938 (Santa Cruz Biotechnology, INC.). Β-actin monoclonal antibody (sc-47778; Santa Cruz Biotechnology) was employed as a loading control. The membranes were then incubated with secondary antibodies (rats anti-rabbit IgG-HRP: sc-2357, Santa Cruz Biotechnology), and protein bands were visualized by x-ray film using enhanced chemiluminescence (ECL) detection reagent based on the manufacturer instructions (Amersham Pharmacia Biotech, Piscataway, NJ, United States) (Mehranfard et al., 2020).

### Statistical analysis

Results are reported as mean ± standard error of the mean (Mean±SEM). The data were analyzed by GraphPad Prism 8 statistical software using One-way ANOVA analysis followed by the post hoc Tukey test. The significance level was set at p<0.05. The Kolmogorov-Simonov normality test was also used.

## Results

### Physicochemical properties of honey

The components of honey in this study included glucose 33.92 g%, fructose 36.9 g%, sucrose < 0.02 g%, F/G 1.09, turanose 1.4 g%, maltose 1.06 g%, melezitose 0.25 g%, proline amino acid 569.4 mg/100 g, free acidity 13.2 meq/kg, electrical conductivity of 0.37 mS/cm, and total phenolic content 0.04 mg/ml. The component of honey was analyzed by Isfahan Hourtash Lab.

### The effect of honey on serum glucose in CUMS-induced rats

**Figure 2:**
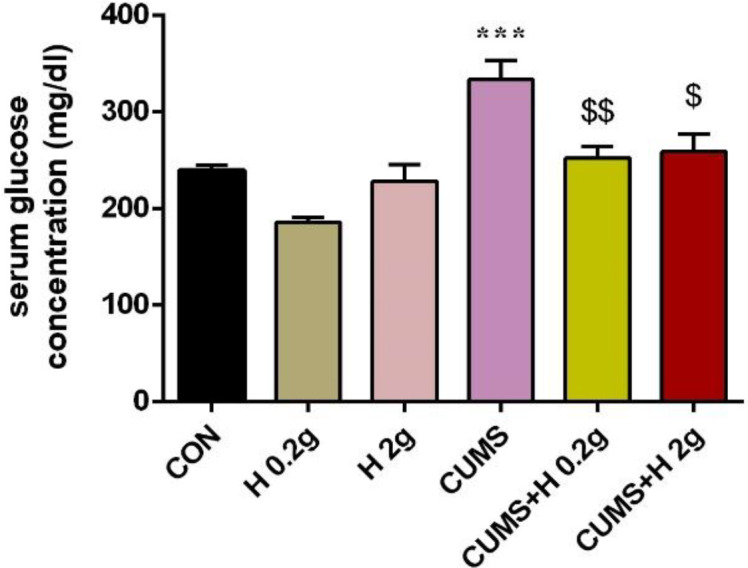
The protective effect of honey on serum level of glucose in stressed groups. ***p<0.001 vs CON group, ^$^p<0.05., ^$$$^p<0.001 vs CUMS group. CON, control; H 0.2 g, honey with dose of 0.2 g; H 2 g, honey with dose of 2 g; CUMS, chronic unpredictable mild stress; CUMS+H 0.2 g, stressed group with dose of 0.2 g honey; CUMS+H 2 g, stressed group with dose of 2 g honey. Data are presented as mean ± SEM.

The results of one-way ANOWA analysis showed that exposure to CUMS in rats, significantly increased the serum glucose concentration as compared to control group (p<0.001). It confirmed the role of chronic stress in development of hyperglycemia. While, treatment with honey (0.2 g and 2 g/kg) could remarkably reduce this hyperglycemia in stressed rats as compared to CUMS group (p<0.01 and p<0.05, respectively). In fact, honey could protect rats against stress-induced hyperglycemia and maintain the blood glucose in control level. But honey treatment in non-stressed groups did not produce a significant difference in glucose levels (Figure 2). 

### The effect of honey on serum insulin in CUMS-induced rats

**Figure 3:**
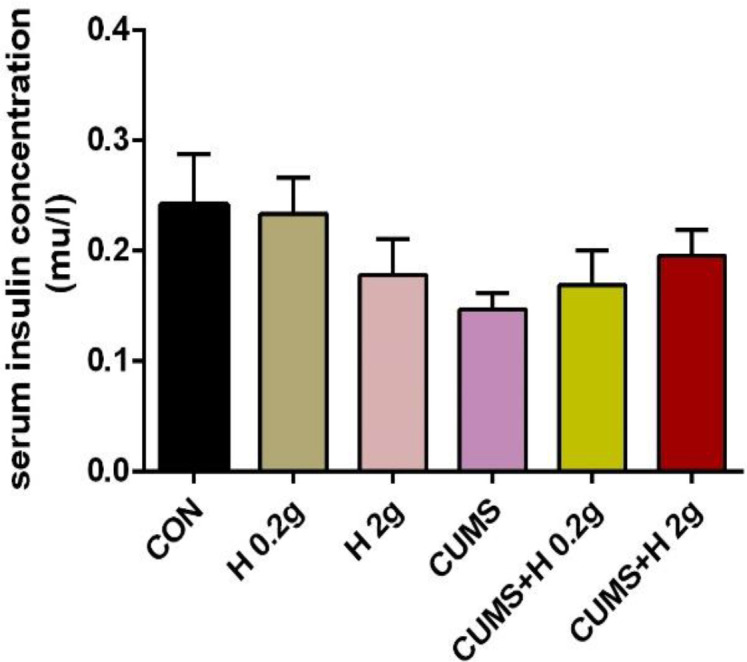
The protective effect of honey on serum level of insulin in stressed groups. CON, control; H 0.2 g, honey with dose of 0.2 g; H 2 g, honey with dose of 2gr; CUMS, chronic unpredictable mild stress; CUMS+H 0.2 g, stressed group with dose of 0.2 g honey; CUMS+H 2 g, stressed group with dose of 2 g honey. Data are presented as mean ± SEM.

Insulin concentration did not show any significant difference among the groups (Figure 3). 

### The effect of honey on serum irisin in CUMS-induced rats

**Figure 4:**
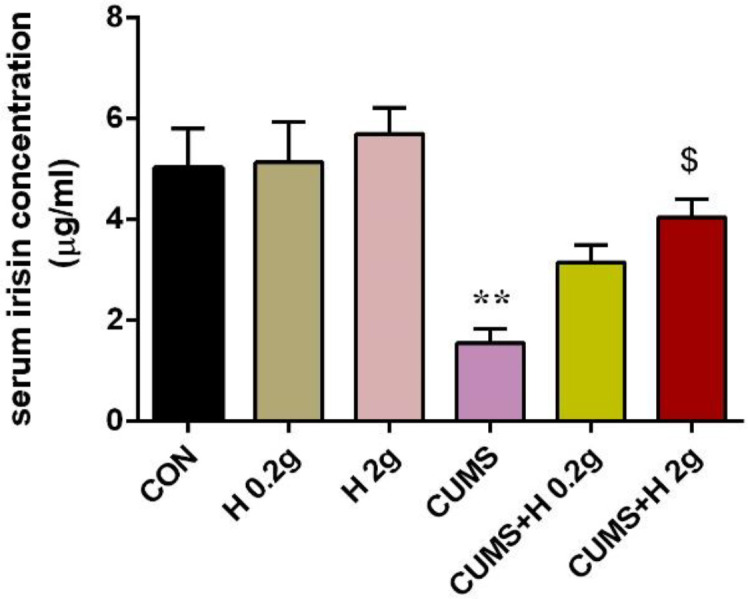
The protective effect of honey on serum level of irisin in stressed groups. **p<0.01 vs CON group, ^$^p<0.05., vs CUMS group. CON, control; H 0.2 g, honey with dose of 0.2 g; H 2 g, honey with dose of 2 g; CUMS, chronic unpredictable mild stress; CUMS+H 0.2 g, stressed group with dose of 0.2 g honey; CUMS+H 2 g, stressed group with dose of 2 g honey. Data are presented as mean ± SEM.

Irisin as an adipomyokine has important role in insulin sensitivity. Circulating irisin levels was measured in all groups the day after the experiment period. In untreated rats that received CUMS, serum irisin concentration significantly decreased in comparison with control group (p<0.01). But after treatment with low and high dose of honey, this result was improved and it was returned approximately to control range. As illustrated in Figure 4, although both doses of honey increased the level of irisin (p<0.05) but this enhancement was non-significant was in the case of the lower dose. On the other hand, honey consumption could not change the serum irisin level in untreated animals (Figure 4).

### The effect of honey on expression of GLUT4 protein in CUMS-induced rats

**Figure 5A:**
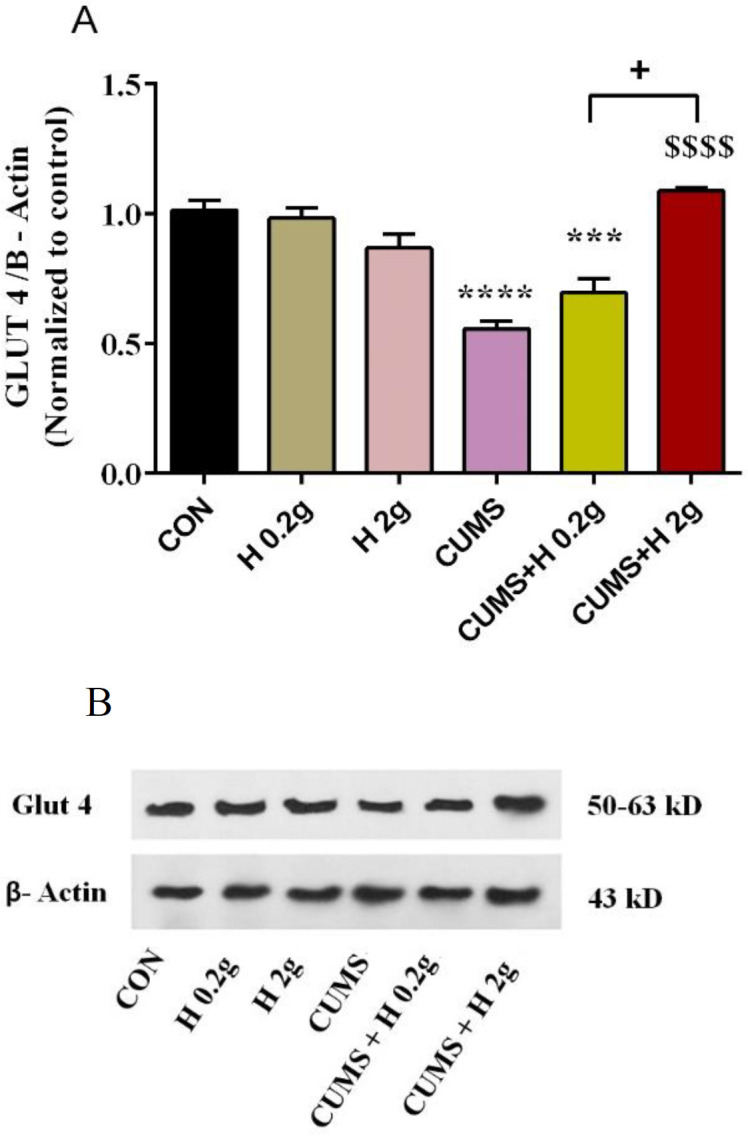
The protective effect of honey on expression level of muscle glut4 protein in stressed groups. ****p<0.0001 and *** p<0.001 vs CON group, ^$$$$^p<0.0001 vs CUMS group, ^+^p<0.0001 difference between CUMS+H 0.2 and H 2 g groups. CON, control; H 0.2 g, honey with dose of 0.2 g; H 2 g, honey with dose of 2 g; CUMS, chronic unpredictable mild stress; CUS+H 0.2 g, stressed group with dose of 0.2 g honey; CUMS+H 2 g, stressed group with dose of 2 g honey. B**.** Representative Western blot assay. Data are presented as mean ± SEM.

The results of Image J densitometries analysis demonstrated a significant reduction in GLUT4 protein expression in skeletal muscle tissue of rats exposed to CUMS when compared to control group (p<0.0001). Administration of high dose of honey (2 g/kg) in stressed group significantly enhanced the decreased level of GLUT4 protein as compared to CUMS group (p<0.0001), while low dose of honey (0.2 g/kg) failed to increase that. Also, there was a marked difference between two treated stress groups, so that high dose of honey increased GLUT4 level more than low dose (p<0. 05). No significant difference was found between the treated groups without stress (Figure 5).

### The effect of honey on lipid profile in CUMS-induced rats

In the present study, the serum concentration of total cholesterol, triglyceride, LDL-c and HDL-c in all groups were detected. As illustrated in Table 1, CUMS in stressed rats enhanced the level of cholesterol and LDL-c (p<0.05-p<0.001), but did not produce significant changes in TG concentration as compared to control group. Treatment of stressed rats with low or high doses of honey could not significantly restore this stress-induced hyperlipidemia. Although, honey with low dose decreased LDL-c level in comparison with control group but it was not able to decrease LDL-c in stressed groups. On the other hand, rats exposed to CUMS indicated a significant reduction in HDL-c levels as compared to control group (p<0.05). Honey administration non-significantly increased it to control group range (Table 1). 

### The effect of honey on body weight in CUMS-induced rats

**Figure 6:**
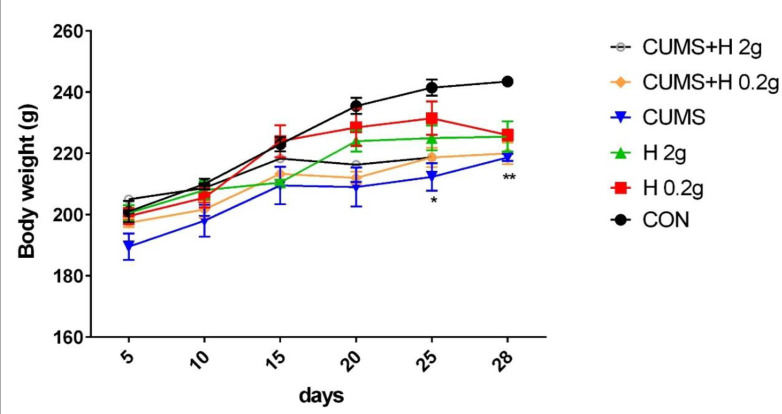
The protective effect of honey on body weight in stressed groups. *p<0.05 and **p<0.01 vs CON group. CON, control; H 0.2 g, honey with dose of 0.2 g; H 2 g, honey with dose of 2 g; CUMS, chronic unpredictable mild stress; CUMS+H 0.2 g, stressed group with dose of 0.2 g honey; CUMS+H 2 g, stressed group with dose of 2 g honey. Data are presented as mean ± SEM.

The analyzed results of body weights in all groups indicated that CUMS significantly reduced body weight in rats in the last days of CUMS procedure as compared to control group (p<0.05-0.01), while treatment with honey in non-stressed or stressed groups produced no significant changes in body weight in comparison with the other groups. In fact, honey could not considerably improve this decrease in body weight of CUMS animals (Figure 6). 

**Table 1:** Serum lipid content in experimental groups

**Groups**	**TC**	**TG**	**LDL-C**	**HDL-C**
CON	42 ± 2.6	41.8 ± 6.1	8.3 ± 0.3	32.2 ± 0.9
H 0.2 g	51.4 ± 1.9	60.4 ± 9.7	7.4 ± 0.24*	26.5 ± 0.6
H 2 g	55.7 ± 1.9	38.3 ± 8.5	9 ± 0.4	30.2 ± 1.4
CUMS	59.7 ± 2.02***	50.3 ± 9.3	9.7 ± 0.3*	24.7 ± 1.3*
CUMS+H 0.2 g	59.6 ± 1.5***	62.5 ± 8.9	9.9 ± 0.3	29.3 ± 2.5
CUMS+H 2 g	58.1 ± 2.5***	48.8 ± 9.3	10.7 ± 0.5	28.3 ± 1.3

*p<0.05 and ***p<0.001 vs CON group. TC, total cholesterol; TG, triglyceride; LDL-C, lowdensity lipoprotein cholesterol; HDL-C, high-density lipoprotein cholesterol; CON, control; H 0.2 g, honey with dose of 0.2 g; H 2 g, honey with dose of 2 g; CUMS, chronic unpredictable mild stress; CUMS+H 0.2 g, stressed group with dose of 0.2 g honey; CUMS+H 2 g, stressed group with dose of 2 g honey.

## Discussion

Honey is a natural sweet food that has various healing properties including anti-inflammatory, antioxidant, antidiabetic, antibacterial, antitumoral and antiviral activities (Chen et al., 2022). Basic and human studies in the last decade have reported that regular consumption of honey improved the hyperglycemia induced by diabetes status (Mohd Ramli et al., 2021; Kadirvelu and Gurtu, 2013). But there is few evidence on the effect of honey on glucose homeostasis and/or molecular mechanisms of insulin action under stress conditions particularly chronic stress. So, in the current study, we focused on the impact of thyme honey on GLUT4 protein expression in muscle tissue, serum concentrations of irisin and glucose, insulin and lipid profile and body weight in rats subjected to chronic unpredictable mild stress. In this study, although CUMS in rats could reduce the body weight in the last days of the experiment, but honey feeding prevented weight loss caused by stress in these rats. In agreement with our findings, CUMS for 5 weeks reduced body weight and the sucrose consumption after doing a sucrose preference test. Reduced weight gain and sucrose intake are two indicators of this model of stress (CUMS) (Liu et al., 2014). 

Furthermore, our results showed that the use of honey in control groups tends to lower the body weight. It seems that using honey in healthy animals reduces the body weight (Omotayo, 2017). Maybe this weight loss is due to a decrease in appetite that was not assayed in this study, and reduction in size and numbers of adipocyte in the mesenteric and intra-abdominal fats (Ugusman et al., 2022). 

As the results of our study showed, CUMS in rats elevated the serum glucose level and impaired lipid profile, especially reduction of HDL-c and elevation of total cholesterol which were similar to other studies conducted on stress (Pereira et al., 2016; Tang et al., 2015b; Tang et al., 2015a). While honey as an anti-diabetic natural agent, interestingly restored lipid profile approximately to normal range in stressed rats. Consistent with our study, Arabmoazzen et al. demonstrated that oral administration of honey 3 times a day for 8 weeks, in male Wistar rats exposed to noise stress for two months, reduced hyperglycemia and generated appropriate changes in serum lipid profile and modulated biomarkers of oxidative stress in the brain (Arabmoazzen et al., 2015). Contrary to our findings, in a human study, receiving supplementation of honey at dose of 60 g/day for 14 days by women who experienced mild stress, could not produce changes in glucose level or total cholesterol (Usman et al., 2020). In this study, we found a non-significant increase in HDL-c level in animals fed with the honey and exposed to stress and even it reached to normal level. In addition, using honey decreased the LDL-c concentration in non-stressed rats, however no marked difference was observed in other lipid levels following honey treatment in rats with or without receiving stress. Similar to our results, Molan reported that honey-fed groups had decreased levels of HDL cholesterol but honey did not affect the other lipids as compared to sucrose fed group (Molan, 2001). In another study, total cholesterol did not significantly change in woman experienced mild stress after receiving the dose of 60 g/day honey for 14 days (Usman et al., 2020). While Arabmoazzen et al. confirmed that honey treatment via gavage method (3 times/day) in rats subjected to noise stress for 8 weeks elicited a remarkable improvement in lipid profile (total cholesterol, TG, LDL, and HDL) changed by chronic noise stress (Arabmoazzen et al., 2015). Furthermore, several recent studies, animal or human, have demonstrated a marked therapeutic effect of honey on hyperlipidemia produced by diabetes mellitus (Sirisha et al., 2021; Ramli et al., 2018; Bahrami et al., 2009; Mazruei Arani et al., 2019; Al-Waili, 2004; El-Haskoury et al., 2019). Perhaps it can be said that the reason for so many variations observed in stress response to honey treatment is the difference in the kind, dose and/or frequency of used honey, duration of treatment, time, duration or type of stress induction processor, the induction pathways of hyperlipidemia and finally species of study animals which are all effective in lipid profile report. 

Our findings indicated that irisin amount was reduced by CUMS induction in rats while honey treatment of these rats for 28 days, could enhance it to control group level. It seems that this improvement effect of honey to be dose-dependent. This modulatory effect of honey was in the same direction as its effect on GLUT4 protein expression in muscle tissue of rats exposed to stress. Our findings showed that CUMS induction in non-treated rats for 28 days dramatically down-regulated the GLUT4 protein level while honey administration preferably with high dose, significantly inhibited this suppressive of CUMS on GLUT4 expression and maintained it at normal level. However honey in non-stressed rats did not produce significant changes in expression of muscle GLUT4. All these results suggest a protective effect of honey consumption on metabolic outcomes induced by chronic stress. In confirmation of our study, Sánchez-Tapia et al. and Hashim et al. indicated a lowered serum glucose level, an increase in phosphorylation of IRS-tyrosine and AKt and adipose tissue GLUT4 overexpression in rats fed with high-fat diet following long-term honey consumption (4 months), indicating an essential role of honey in better insulin signaling (Hashim et al., 2021; Sánchez-Tapia et al., 2019). Also, another study reported that honey maintained glucose homeostasis, decreased insulin resistance via modulating IRS/PI3K/AKT signaling pathways (Chen et al., 2022). 

It is documented that polyphenols presence in honey are responsible for improving the insulin signaling. The other component of honey is fructose, as an abundant monosaccharide in honey, which reduces blood glucose level due to its prolonged interaction with intestinal receptor (Erejuwa et al., 2012; Hashim et al., 2021). So, many therapeutic impacts of honey are attributed to its bioactive ingredients. 

Irisin as a new adipomyokine was explored in 2012 (Perakakis et al., 2017). It has been linked to insulin action in adipose or muscle tissues. It increases the insulin sensitivity and improves the insulin signaling pathways (Xin et al., 2016; Yang et al., 2015) and finally it attenuates diabetes/stress-induced hyperglycemia. GLUT4 as a key regulator for glucose homeostasis, is highly expressed in skeletal muscle and adipose tissue (Lee et al., 2015). There are conflicting studies about the effect of irisin on GLUT4 expression. It was shown that irisin has no effect on GLUT4 gene expression but it elevated glucose uptake via stimulating GLUT4 translocation from cytoplasm to cell surface (Lee et al., 2015), while in another study, it was found that irisin increased the expression of GLUT4 in primary human skeletal muscle cell (Huh et al., 2014). On the other hand, irisin can reduce lipid profile via increasing lipid metabolism particularly in insulin resistance state (Gamas et al., 2015). So, our results suggested that honey may modulate dyslipidemia and glucose dysregulation produced by CUMS, via an enhancement in circulating irisin and GLUT4 protein expression in skeletal muscle as a major organ in maintaining glucose homeostasis. 

In summary, our results demonstrated that CUMS in rats for 28 days elicited hyperglycemia, mild dyslipidemia, reduction of irisin secretion, down-regulation of GLUT4 protein expression; honey feeding protectively prevented all these metabolic disorders induced by CUMS. Therefore, it can be conclude that honey increased capacity for glucose transport via increasing irisin secretion and up-regulation of GLUT4 protein level in insulin-sensitive tissues such as skeletal muscle which led to maintaining glycemic control under chronic unpredictable mild stress condition. Further studies are needed to investigate the effect of honey on insulin signaling pathways and insulin resistance or glucose homeostasis following CUMS. 
